# Naringenin as a Multifunctional Ingredient Targeting Pathways Associated With Skin Inflammaging: Preclinical and Clinical Evidence

**DOI:** 10.1111/jocd.71167

**Published:** 2026-09-06

**Authors:** Taylor Oswald, Zixuan Shao, Nicholas Brideau

**Affiliations:** ^1^ Debut Biotechnology, Inc San Diego California USA

**Keywords:** anti‐aging, anti‐inflammatory, cell culture, clinical trials, flavonoids, genetic analysis, hallmarks of aging, inflammaging

## Abstract

**Background:**

Inflammaging, a sustained state of low‐level inflammatory activity with age, is a driving factor in age‐related changes in skin structure, function, and appearance. By perturbing fundamental biological pathways, inflammaging is a relevant target in skincare and anti‐aging research.

**Aim:**

The objective of this study was to investigate the anti‐inflammatory and anti‐aging potential of naringenin, a natural flavonoid, using a combination of mechanistic in vitro analyses and a controlled human clinical evaluation.

**Methods:**

The activities and mechanism of action of naringenin were tested on normal human epidermal keratinocytes and in complementary acellular assays. A placebo‐controlled clinical study was conducted to assess the effects of topical naringenin on multiple skin parameters.

**Results:**

Transcriptomic analysis was applied as a discovery‐driven strategy to characterize the molecular responses induced by naringenin. Naringenin was found to have anti‐inflammatory properties, including downregulation of proinflammatory pathway genes, a significant reduction in biomarkers interleukin‐6 (IL‐6) and tumor necrosis factor‐α (TNF‐α) in keratinocytes, and a reduction in visible redness in clinical trial participants. In addition to its anti‐inflammatory activity, naringenin exhibited a multimodal anti‐aging profile, including gene expression changes consistent with modulation of skin aging, radical‐scavenging capacity, inhibition of elastase activity, and reduced protein glycation. Topical application led to statistically significant improvements in multiple skin attributes.

**Conclusion:**

These findings identify naringenin as a multifunctional bioactive ingredient with anti‐inflammaging properties relevant to skincare. The anti‐inflammaging function of naringenin is further supported by clinical outcomes showing improved wrinkle appearance, skin elasticity, and hydration following topical use.

## 1 Introduction

The phenomenon of inflammaging, the persistent low‐grade systemic inflammation that occurs as we age, is considered one of the major biological Hallmarks of Aging [1, 2, 3]. It is marked by the release of proinflammatory cytokines, other senescence‐associated secretory phenotype (SASP) factors, and elevated production of reactive oxygen species [4, 5, 6]. Multiple proinflammatory cytokines, including IL‐6, IL‐8, and TNF‐α, are components of the SASP‐related profile and modulate gene expression, leading to senescent cell accumulation and impaired regenerative capacity [7, 8]. This chronic immune activation underlies many age‐related pathologies, including the deterioration of skin health and appearance. In skin, inflammaging manifests as impaired barrier function, extracellular matrix (ECM) degradation, and reduced regenerative capacity [9]. Compromised epidermal barrier function increases vulnerability to external damage, leading to dryness, rough texture, and uneven tone [10, 11]. Degradation of collagen and elastin clinically results in reduced skin resilience and the appearance of wrinkles, while impaired regeneration from senescent cell accumulation slows the skin's ability to repair itself [12, 13]. Collectively, these processes create a cycle in which inflammation contributes to cellular and tissue dysfunction, resulting in the visible acceleration of skin aging.

Flavonoids are a diverse class of natural, plant‐derived molecules with active properties [14]. These compounds are recognized for their capacity to counter oxidative stress and modulate inflammatory pathways, positioning them as promising candidates for skincare [15, 16, 17, 18]. Flavonoids can be distinguished by chemical structure and further classified into smaller groups, such as flavonols (quercetin, myricetin), flavones (luteolin, apigenin), and flavanones (naringenin, hesperetin) [15, 19, 20]. Naringenin is one of the most common and abundant flavanones and is primarily found in citrus fruits such as grapefruits, lemons, and oranges [21]. It has been investigated for potential therapeutic applications across multiple health conditions, including metabolic health (diabetes, obesity), cardiovascular diseases (hypertension, atherosclerosis), neurodegenerative diseases (Alzheimer's, Parkinson's), organ fibrosis, and several types of cancer [22, 23, 24]. The protective effects of naringenin also extend to the skin, where it has been shown to inhibit LPS‐induced inflammation in human dermal fibroblasts and improve oleic acid‐induced skin damage in mice [25, 26]. As an antioxidant, naringenin can reduce reactive oxygen species, including those induced by UV‐B radiation, in both human fibroblasts and mouse models [27, 28].

Across these studies, the reported activities of naringenin have been linked primarily to modulation of oxidative stress and inflammatory signaling. These mechanisms support a role for naringenin in influencing key biological processes and the health of the skin. Reactive oxygen species (ROS) from sources such as UV‐B radiation act as upstream triggers to activate redox‐sensitive transcription factors, activator protein‐1 (AP‐1) and nuclear factor kappa‐B (NF‐κB) [29]. Activation of these pathways drives the transcription of proinflammatory mediators and matrix metalloproteases, promoting chronic inflammation and ECM degradation, leading to skin aging [30]. Cells have regulatory pathways to mitigate these damaging processes, including nuclear factor erythroid‐2‐related factor 2 (Nrf2) and sirtuin 1 (SIRT1) signaling [31, 32]. Naringenin has been reported to reduce ROS, suppress NF‐κB signaling cascades, and boost both Nrf2 and SIRT1 expression to restore redox homeostasis and rebalance inflammatory signaling in human fibroblast and murine systems [25, 26, 27].

Despite evidence for antioxidant and anti‐inflammatory activity, the role of naringenin in skin inflammaging remains incompletely defined. Previous studies have largely focused on individual mechanisms or single model systems, with limited characterization of naringenin's wider effects on the processes underlying inflammation and skin aging. To address this gap, the present study investigated the broader biological activity of naringenin, integrating transcriptomic analysis, complementary in vitro assays, and controlled clinical evaluation.

## 2 Materials and Methods

### 2.1 Naringenin

Naringenin (INCI: Naringenin, IUPAC: (2*S*)‐4′,5,7‐Trihydroxyflavan‐4‐one) was sourced from Debut Biotechnology Inc. (DermCeutical Naringenin, San Diego, CA, USA) as an off‐white powder with a purity of 98% (*w/w*) by qNMR analysis (Figure S1). Naringenin was biomanufactured via microbial fermentation using engineered *E. coli* strains, followed by a proprietary extraction & purification process.

### 2.2 Cell Culture and Treatments

Normal Human Epidermal Keratinocyte (NHEK) cells (ATCC PCS‐22‐011 lot 80 304 222) were grown in EpiLife Medium (Thermo Fisher Scientific, Waltham, MA, USA) supplemented with human keratinocyte growth supplement (HKGS) and gentamicin (Bio Basic, Amherst, NY, USA). Cryopreserved cells were grown until 80% confluency and passaged up to three times.

### 2.3 Cell Viability Assay

Cell viability was determined using the MTT assay, which assesses cellular metabolic activity. Metabolically active cells reduce the tetrazolium compound 3‐(4,5‐dimethylthiazol‐2‐yl)‐2,5‐diphenyltetrazolium bromide (MTT) to formazan, producing a measurable colorimetric signal. Assays were performed using a commercially available kit (Sigma‐Aldrich, St. Louis, MO, USA) according to the manufacturer's instructions. Cytotoxicity was defined as cellular viability < 80% relative to the untreated control. Statistical analysis was performed using a one‐way ANOVA followed by Dunnett's post hoc test to compare the four treatment concentrations against the untreated control group. Differences were considered statistically significant at *p* < 0.05.

### 2.4 Transcriptomic Analysis

NHEK cells were grown as described above and treated with naringenin (Debut Biotech) or DMSO (Sigma‐Aldrich) control for 24 h (*N* = 3). Following treatment, cells were detached from cell culture plates with TrypLE (Gibco, Thermo Fisher Scientific), rapidly cryopreserved, and stored at −80°C until further processing. Frozen cell pellets were transported on dry ice to an external service provider (Genewiz, South Plainfield, NJ, USA) for total RNA extraction, library construction, and next‐generation sequencing. Messenger RNA was enriched via poly‐A selection during library preparation, and unique molecular identifiers (UMIs) were incorporated to enable removal of duplicate reads. Sequencing was performed using paired‐end reads (150 bp) with an average depth of 30 million reads per sample. Processed reads were aligned to the human reference genome (GRCh38) and annotated transcriptome (Gencode v44) using STAR (version 2.7.11a). Gene counts were determined using featureCounts based on the mapping results produced by STAR (version 2.0.6). DESeq2 (version 1.44.0) was used to identify statistically significant differentially expressed genes. Pathway‐level changes were evaluated by enrichment analysis of Kyoto Encyclopedia of Genes and Genomes (KEGG) pathways using the GSEA algorithm as implemented in ClusterProfiler (version 4.12.0). In the GSEA algorithm, genes were ranked by their differential expression in naringenin‐ vs. DMSO‐treated samples, and this ranking was used to assess whether genes belonging to specific pathways were enriched toward either end of the ranking. Pathways with an adjusted *p*‐value below 0.05 were considered statistically significant, with the sign of the normalized enrichment score (NES) indicating the direction of pathway regulation (positive for up‐regulation, negative for down‐regulation). Visualization of selected KEGG pathways with integrated gene expression data was generated with Pathview (version 1.44.0), which overlays differential expression data onto KEGG pathway diagrams to facilitate interpretation of pathway regulation.

### 2.5 Antiaging Assays

#### 2.5.1 Radical Scavenging

Antioxidant activity was assessed using the DPPH radical scavenging assay. This method quantifies the reduction of the stable radical 2,2‐diphenyl‐1‐picrylhydrazyl (DPPH), which is accompanied by a decrease in absorbance. Stock solutions were prepared on the day of analysis and included 200 μM DPPH in ethanol (Cayman Chemical 14 805), 400 μM Trolox (Cayman Chemical 10 011 659) for standard curve generation, and 0.01% (w/w) *L*‐Ascorbic Acid as a positive control; ethanol served as the negative control. Reactions combined equal volumes of DPPH solution and the test sample, followed by incubation at 25°C in the dark for 30 min. Absorbance was recorded at 517 nm using a plate reader (Spectramax M2, Molecular Devices, San Jose, CA, USA).

#### 2.5.2 Elastase Inhibition

Stock solutions were prepared as follows: 0.1 M Tris–HCl (pH 7.5) as assay buffer, human neutrophil elastase (HNE) (1 U/mL in assay buffer), MeO‐Suc‐Ala‐Ala‐Pro‐Val‐pNA (MAAPVN) at 1.0 mM in DMSO as the enzyme substrate, oleanolic acid (200 μg/mL) as a positive control. DMSO served as the solvent for the test compound and as the negative control. For the enzymatic assay, working solutions of HNE and the test compounds were combined and preincubated at 25°C for 10 min. MAAPVN was then added, and the mixture was incubated at 37°C for 1 h. The enzymatic activity was determined by measuring the release of *p*‐nitroaniline at 405 nm using a microplate reader (SpectraMax M2, Molecular Devices).

#### 2.5.3 Glycation Inhibition

Stock solutions were prepared using 67 mM phosphate buffer (pH 7.4) containing 3 mM sodium azide for assay buffer, bovine serum albumin (BSA) at 20 mg/mL, glucose at 270 mg/mL, and aminoguanidine (1.1 mg/mL) as a positive control. Phosphate buffer or DMSO was used as the solvent for test compounds and as the negative control, as appropriate. Reactions combined 50 μL of BSA, 50 μL of the test article or control, and 50 μL of either glucose or buffer. Samples were incubated at 40°C for 7 days in the dark to allow non‐enzymatic glycation to occur. Formation of advanced glycation end‐products (AGEs) was quantified by fluorescence on a microplate reader (SpectraMax M2, Molecular Devices) using 360 and 420 nm as the excitation and emission wavelengths.

### 2.6 Anti‐Inflammatory Activity

NHEK cells were cultured as described above and seeded at a density of 7.5–10.0 × 10^3^ cells/cm^2^ (3.6–4.8 × 10^4^ cells/mL in 2 mL media per well; 6‐well plate, 9.6 cm^2^ growth area). Cells were maintained at 37°C for 48–72 h to allow attachment and stabilization. Culture medium was then replaced with fresh, pre‐warmed medium, and cells were treated with an inflammatory cocktail consisting of lipopolysaccharide (LPS, 2.0 μg/mL from *Escherichia coli* O26:B6) and polyinosinic‐polycytidylic acid (poly(I:C), 12.5 μg/mL). After one hour of inflammatory stimulation, test compounds were added, and cells were incubated for an additional 24 h. Conditioned media was collected for analysis of inflammatory mediators. Levels of TNF‐α and IL‐6 were quantified using commercially available ELISA kits (ELH‐TNFa, ELH‐IL6‐1, RayBiotech, Peachtree Corners, GA, USA) according to the manufacturer's instructions. Absorbance was measured using a plate reader (SpectraMax M2, Molecular Devices).

### 2.7 Clinical Trials

#### 2.7.1 Ethical Conduct

This study was conducted in accordance with the intent and purpose of Good Clinical Practice regulations described in Title 21 of the U.S. Code of Federal Regulations (CFR) and the Declaration of Helsinki.

#### 2.7.2 Subject Information and Consent

This study was conducted in compliance with CFR Title 21, Part 50 (Informed Consent of Human Subjects). Informed Consent was obtained from each subject in the study and documented in writing before their participation, including for the publication of their photographs and relevant clinical information. A copy of the Informed Consent was provided to each subject.

#### 2.7.3 Study Design and Controls

Sixty‐eight male and female volunteers aged 37–69 years participated in an 8‐week, randomized, double‐blind, placebo‐controlled clinical study. Subjects applied the assigned formulation to the facial area twice daily. The active formulation contained 0.45% naringenin, while the placebo formulation was identical in composition but lacked the active ingredient. Clinical and instrumental evaluations of facial skin parameters were performed at baseline, after 4 weeks of treatment, and at study completion (8 weeks).

Participants were eligible for enrollment if they met all of the following criteria: (1) Male and female adults aged 35–69 years (inclusive), in generally good health, as determined by medical history (no physical examination required). (2) Willingness and ability to comply with all study procedures, including product application, scheduled visits, and study duration (8 weeks). (3) Presence of fine lines and wrinkles, defined as a score of ≥ 5 on the investigator‐assessed facial wrinkle scale. (4) Presence of deep wrinkles, defined as a score of ≥ 7 on the investigator‐assessed facial wrinkle scale. (5) Presence of facial erythema, defined as a score of ≥ 5 (moderate visible redness) for qualification purposes. (6) Ability to read, understand, and sign the informed consent form and photo release authorization. (7) Demonstrated ability to follow study instructions and apply the investigational product as directed. (8) Regular use of a facial moisturizer prior to study enrollment. (9) Representation of all skin types (normal, oily, dry, and combination). (10) Inclusion of subjects with darker skin phototypes, with at least five participants classified as Fitzpatrick skin types IV–VI.

Participants were excluded if they met any of the following criteria: (1) Failure to meet any of the inclusion criteria. (2) Pregnancy, planned pregnancy, or lactation during the study period. (3) Known sensitivity or allergy to facial skincare products or any component of the investigational formulation. (4) Use of systemic or topical anti‐inflammatory agents within 5 days prior to baseline, with the exception of over‐the‐counter acetaminophen, ibuprofen, or aspirin. (5) Unwillingness to refrain from systemic or topical anti‐inflammatory agents (with the same OTC exceptions) for the duration of the study. (6) Participation in another facial clinical study within 14 days prior to study initiation. (7) Presence of any facial piercings. (8) Presence of facial or neck tattoos, with the exception of thin eyeliner confined to the upper lash line (no winged eyeliner). (9) Unwillingness to remove facial jewelry (e.g., earrings) prior to standardized photography.

#### 2.7.4 Clinical Data Collection

Standardized facial photographs were acquired by trained technicians using a high‐resolution imaging system (VISIA CR 2.2, Canfield Scientific, Farfield, NJ). Digital images were processed and quantified with image analysis software (ImagePro, MediaCybernetics, Bethesda, MD) to assess changes in wrinkle‐related parameters. Fine lines and deep wrinkles were identified under controlled illumination conditions, including standard and fixed‐angle cross‐polarized lighting, and quantified as mean area values. Reductions in these values were interpreted as improvements in wrinkle appearance, whereas increases indicated deterioration.

Facial erythema was evaluated by trained technicians using a standardized ordinal scale ranging from 0 to 9, where lower scores reflected minimal redness and higher scores indicated increasing redness severity. A decrease in erythema score was considered an improvement, while an increase represented worsening.

Additional skin properties were further assessed using noninvasive instrumentation. Skin elasticity was measured on the facial area using a Cutometer (Courage + Khazaka, Germany), with elastic recovery quantified by the R2 parameter; higher R2 values indicated improved elasticity. *Stratum corneum* hydration was evaluated using a Corneometer (Courage + Khazaka, Germany), with increases in measured values interpreted as enhanced skin hydration and decreases as reduced moisture content.

### 2.8 Statistical Analysis

For in vitro experiments, statistical comparisons were conducted between test articles and their corresponding solvent controls. Differences were considered statistically meaningful when the probability value was ≤ 0.05.

For clinical evaluations, outcome measures at each post‐treatment time point were compared to the individual baseline values for each subject. Changes from baseline were analyzed using either the Wilcoxon Signed‐Rank test or Student's *t*‐test, as appropriate. Statistical significance was defined as a *p*‐value ≤ 0.05. Comparative analyses were performed both within treatment groups across time points and between treatment and control groups at corresponding assessments.

Subjects with improvement from baseline (%) represents the percentage of subjects whose outcome measure improved relative to their baseline.

### 2.9 Artificial Intelligence Use

Generative AI tools (OpenAI ChatGPT, Anthropic Claude, Google Gemini) were used to assist with language editing and improve readability throughout the manuscript. These tools were not used to generate or interpret data, or to produce scientific content. All AI‐assisted text was reviewed, edited, and verified for accuracy by the authors, who take full responsibility for the content of the manuscript.

## 3 Results

### 3.1 Naringenin Is an Effective Anti‐Inflammatory Bioactive Molecule

#### 3.1.1 Downregulation of Inflammation Genes in Keratinocytes

Gene expression profiling can serve as a valuable tool for skin health, revealing molecular changes associated with known biological pathways linked to skincare outcomes. For example, understanding shifts in specific gene expression patterns in healthy vs. stressed cells could enable the discovery of novel bioactive ingredients to restore and maintain skin function. In this study, we used transcriptomic analysis to explore the effects of naringenin on skin biology.

**FIGURE 1:**
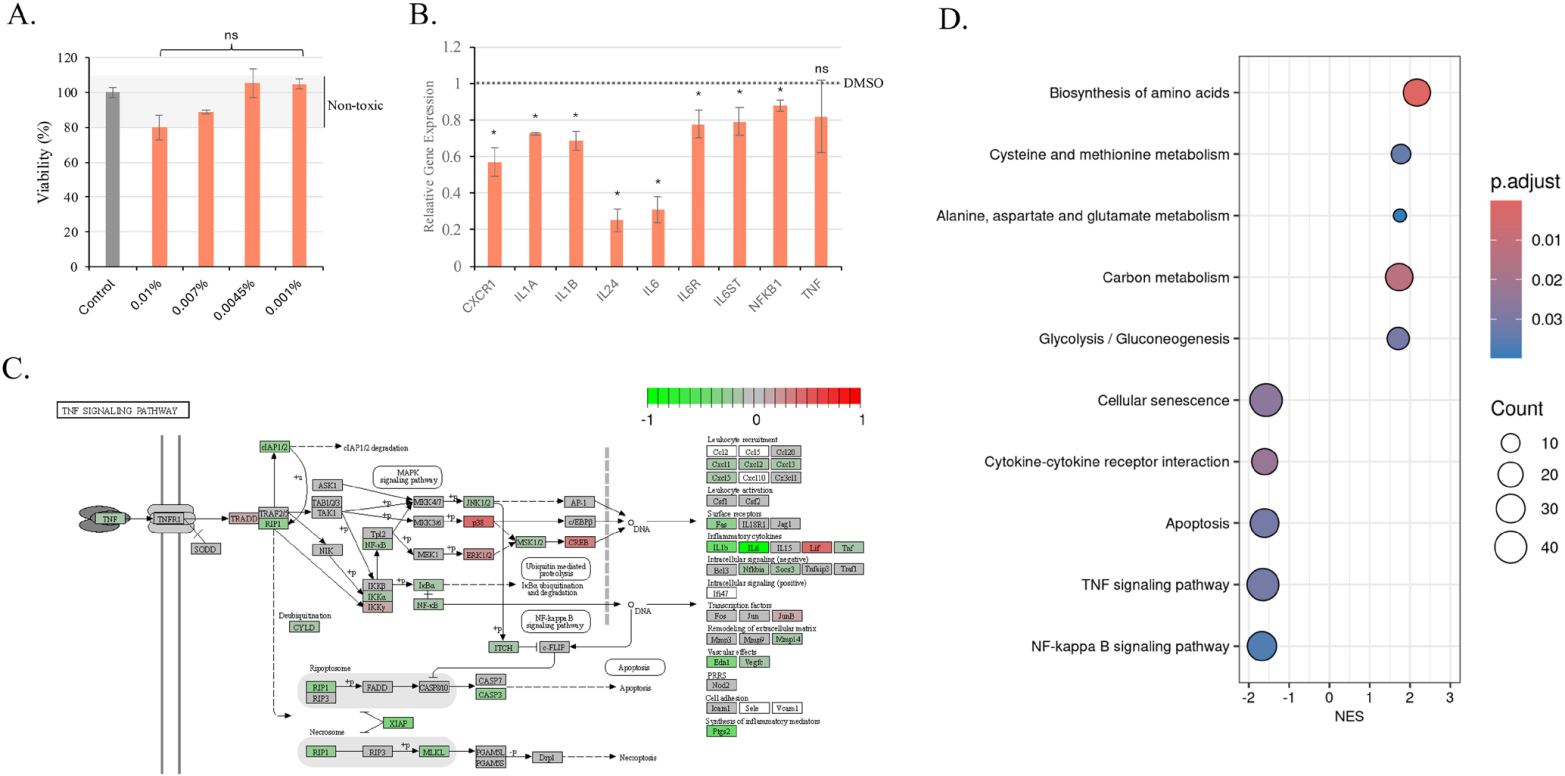
Naringenin regulates the expression of multiple biological pathways, including many associated with inflammation. (A) Naringenin is not cytotoxic in the range of tested concentrations using a predefined cell viability threshold of > 80% relative to control. Data are presented as a mean +/− SD (*n* = 3). All treatment groups showed no statistically significant differences compared to the untreated control (ns = not significant). (B) Example genes associated with inflammation that are downregulated by naringenin in keratinocytes. *p < 0.01. (C) Detailed map of the TNF signaling pathway (from the Kyoto Encyclopedia of Genes and Genomes) superimposed with the modulation of individual genes by naringenin. (D) A visual representation of Gene Set Enrichment Analysis emphasizing the biological processes affected by naringenin. NES = Normalized Enrichment Score.

Naringenin was first tested at multiple concentrations to ensure the use levels were not cytotoxic to keratinocytes. The range of concentrations tested in vitro corresponds to 0.1%–1.0% in a formulated product and is within the solubility and stability limits of the molecule. Cytotoxicity analysis using the MTT assay shows that naringenin is not considered cytotoxic at any tested concentration (Figure 1A). Although all concentrations up to 0.01% met the threshold for non‐toxicity, the concentration of 0.0045% was selected as the appropriate dose for gene expression analysis as it represents the highest tested concentration with no observable effect on viability.

NHEK cells were then treated with test articles and harvested for RNA sequencing to identify changes in gene expression. RNA‐seq results revealed that naringenin down‐regulated the gene expression of a group of proinflammatory cytokines and mediators (Figure 1B). Pathway evaluation of the gene expression changes revealed that naringenin downregulated established proinflammatory cascades, including *TNF‐α* and *NF‐κB* (TNF‐α pathway shown in Figure 1C). In addition to TNF‐α and NF‐κB, cytokine‐cytokine receptor interaction, a biological process central to immune signaling, was downregulated in naringenin‐treated cells (Figure 1D).

#### 3.1.2 Reduction of Key Inflammatory Proteins in Keratinocytes

Gene expression profiling of keratinocytes following naringenin treatment indicated a downregulation of proinflammatory signaling. We therefore sought to connect transcriptomics to other biomarkers of aging to verify our approach. Aging is linked to elevated circulating cytokines and proinflammatory markers, with IL‐6 and TNF‐α notably increased in inflammaging phenotypes [33]. To test whether the reduction in *IL‐6* and *TNF* mRNA results in a reduction in their encoded proteins, we tested for inflammatory proteins in conditioned media from keratinocytes.

**FIGURE 2:**
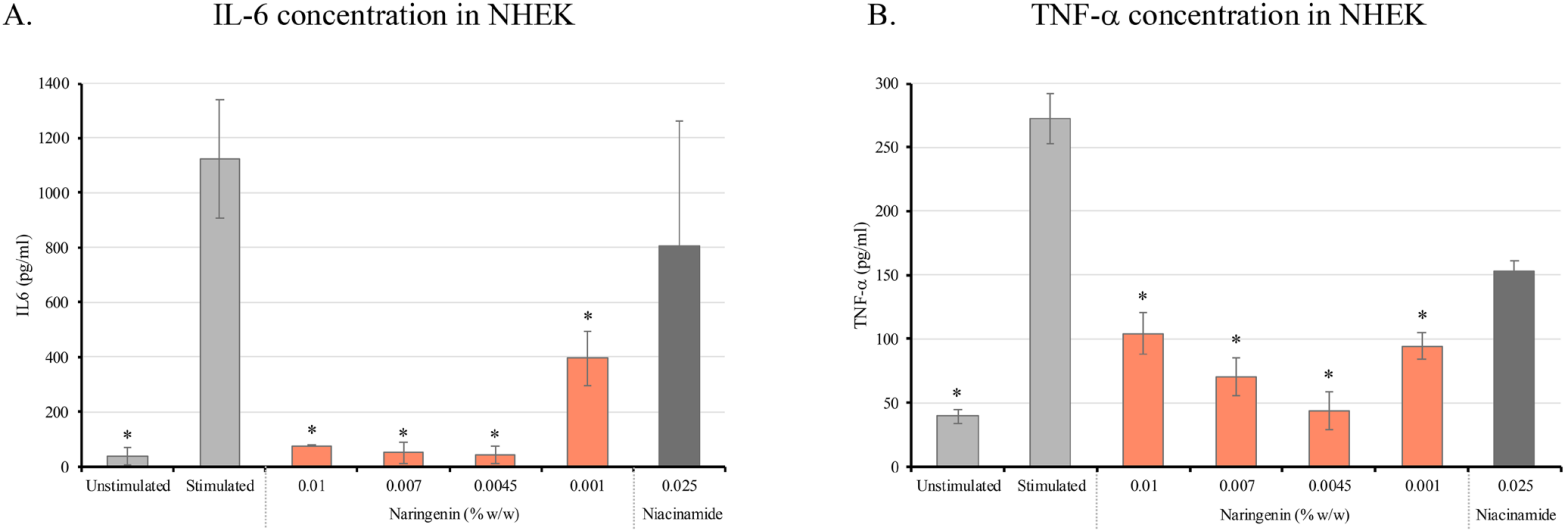
Naringenin has anti‐inflammatory activity, as shown by reductions in the inflammation markers IL‐6 (A) and TNF‐α (B). An asterisk (*) indicates the test product concentrations (% *w/w*) that showed statistically significant differences compared to stimulated controls. Unstimulated and stimulated controls are colored gray, naringenin treatments orange, and niacinamide benchmark comparison charcoal. Data are plotted as mean +/− SD, with a statistically significant threshold of *p* < 0.05.

NHEK cells stimulated with the LPS‐poly(I:C) inflammatory cocktail (positive control) exhibited significantly elevated IL‐6 and TNF‐α levels relative to untreated controls, confirming successful induction of an inflammatory response (Figure 2, gray bars in A, B). Stimulated cells treated with naringenin at 0.0045% demonstrated potent anti‐inflammatory effects, reducing IL‐6 by 96% and TNF‐α by 78%. Treatment with naringenin normalized both markers to levels similar to those of unstimulated cells and outperformed the widely used anti‐inflammatory skincare ingredient niacinamide (Figure 2, charcoal bars).

#### 3.1.3 Clinical Performance of Naringenin to Reduce the Appearance of Inflammation

The gene expression profile of skin cells following naringenin treatment indicated functional regulation of pathways associated with skin inflammaging. Consistent with these transcriptomic findings, additional preclinical studies demonstrated anti‐inflammatory activity by reducing key inflammatory cytokines. To further validate the preclinical observations in a clinically relevant setting, a human clinical study was conducted to evaluate the effects of naringenin on the appearance of skin inflammation. The study compared the use of a simple base formulation containing either a placebo or naringenin (0.45%) at baseline, 4‐week, and 8‐week timepoints.

Inflammation of the skin is frequently associated with visible erythema (redness), resulting primarily from vasodilation and increased blood flow in the affected tissue [34]. This response is a hallmark of various inflammatory skin conditions and serves as an observable marker of immune activation. Skin redness was assessed at baseline and after 4 and 8 weeks of product application for the test articles. A decrease in the score indicates an improvement; the summary is presented in Table 1.

**TABLE 1:** Summary of skin redness evaluation.

Article	Time	Mean score ± SD	*p*	Mean change from baseline (%)	Subjects with improvement from baseline (%)
Placebo	Baseline	5.5 ± 0.6	—	—	—
	4 weeks^a^	5.2 ± 0.6	< 0.001	−5.5	30
	8 weeks^a^	4.7 ± 0.6	< 0.001	−14.5	70
Naringenin	Baseline	6.9 ± 0.9	—	—	—
	4 weeks^a^	6.7 ± 1.0	0.003	−2.9	24
	8 weeks^a^	6.0 ± 1.0	< 0.001	−13.0	88

^a^ Statistically significant difference from baseline, *p* ≤ 0.05.

**FIGURE 3:**
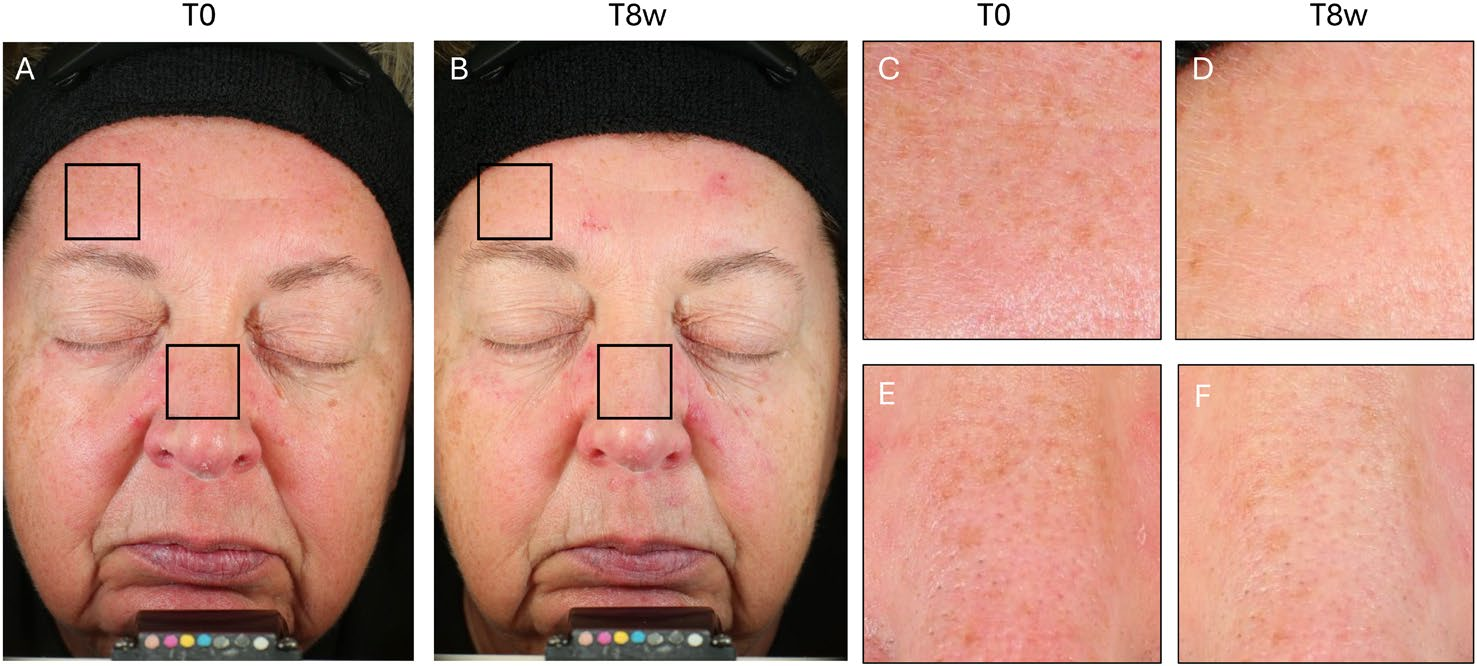
An example clinical study participant demonstrating a reduction in visible redness following naringenin topical treatment. (A) Baseline time zero full face image, and (B) eight‐week timepoint full face image. The images in C and E provide a more detailed view of the black boxes highlighted in A, while the images in D and F offer a more detailed view of the black boxes shown in B.

For the naringenin subjects, measurements at 4 and 8 weeks compared to baseline showed mean change improvements of 2.9% and 13.0%. These improvements were significant, with 24% of the subjects showing improvement at 4 weeks, and 88% showing improvement at 8 weeks of naringenin use. The reduction of redness can be seen in an example subject in Figure 3. Black boxes highlight areas of noticeable improvement following 8 weeks of naringenin treatment.

Together, the reduction in inflammation pathway gene expression and proinflammatory cytokine signaling in skin cells, along with the visible redness in subjects following naringenin topical treatment, all indicate that naringenin is a potent anti‐inflammatory molecule.

### 3.2 Naringenin Targets Multiple Mechanisms of Aging

#### 3.2.1 Gene Expression Modulation in Keratinocytes

While naringenin significantly reduced gene expression across multiple inflammation‐related pathways, the observed changes were not limited to the inflammatory response. Naringenin also downregulates genes directly linked to aging, as evidenced by a reduction in broad cellular senescence and apoptosis pathways (Figure 1D). Consistent with the downregulation of replicative arrest pathways, we also observed an upregulation in gene expression for a group of biological processes related to metabolic health. Carbon, amino acid, and energy metabolism all influence healthy aging through the regulation of oxidative stress and redox balance.

#### 3.2.2 Pre‐Clinical Anti‐Aging In Vitro Validation

To substantiate additional anti‐aging bioactivities of naringenin, a series of preclinical in vitro assays was conducted. These assays were designed to evaluate naringenin's antioxidant capacity as well as its ability to inhibit elastase and the formation of non‐enzymatic protein glycation products. The results support more general anti‐aging properties predicted by gene expression and complement the anti‐inflammatory activity.

##### 3.2.2.1 Radical Scavenging Activity

**FIGURE 4:**
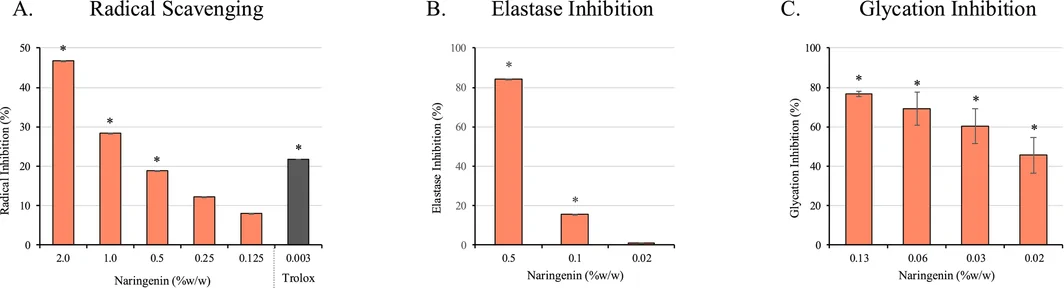
Naringenin shows activity in multiple in vitro anti‐aging assays. (A) DPPH radical scavenging assay, (B) Human Neutrophil Elastase inhibition assay, (C) Advanced Glycation End products assay. An asterisk (*) indicates the naringenin concentrations (%w/w) that showed statistically significant differences compared to solvent controls. Data are plotted as mean +/− SD, with a statistically significant threshold of *p* < 0.05.

ROS are highly reactive chemical species formed in the skin as a result of environmental stressors such as UV exposure and air pollution [35]. In a skincare context, excessive ROS are detrimental because they induce oxidative damage, which accelerates skin aging, promotes inflammation, and compromises structural proteins and cellular components [36]. To counteract these effects, skincare formulations incorporate antioxidant compounds capable of scavenging free radicals. Antioxidant capacity can be evaluated using radical scavenging assays such as the DPPH (2,2‐diphenyl‐1‐picrylhydrazyl) assay, which quantifies an ingredient's ability to neutralize free radicals. Naringenin's radical scavenging activity was measured using the DPPH assay, with Trolox as a positive control. Naringenin concentrations ranging from 0.5% to 2.0% demonstrated significant concentration‐dependent radical scavenging activity compared with the solvent control (Figure 4A). The two lower concentrations (0.25% and 0.125%) did not reach statistical significance (*p* = 0.06), although a trend toward increased radical scavenging activity was observed.

##### 3.2.2.2 Elastase Inhibition Activity

Exposure to external stressors, including UV radiation, leads to elevated neutrophil recruitment in the skin and increased activity of neutrophil elastase (HNE). Neutrophil elastase has been shown to activate MMP‐1 and MMP‐2 collagenases in response to low‐dose UV exposure, harming the extracellular matrix and contributing to photoaging and wrinkle formation [37]. The activity of elastase can be further upregulated in the presence of reactive oxygen species [38]. Therefore, inhibition of this enzyme, particularly in combination with antioxidant activity, may help delay wrinkle development and maintain skin integrity. This assay measures the release of 4‐nitroaniline by human neutrophil elastase and can be quantified. Naringenin concentrations of 0.5% and 0.1% significantly inhibited elastase activity compared to solvent controls (Figure 4B). Furthermore, the higher concentration of naringenin shows 84% inhibition of elastase.

##### 3.2.2.3 Glycation Inhibition Activity

AGEs arise from the non‐enzymatic modification of proteins or lipids by sugars, a process that is enhanced during aging and under conditions of oxidative stress [39]. In the skin, AGE accumulation disrupts collagen and elastin integrity, resulting in diminished skin elasticity, increased wrinkle formation, and loss of skin radiance [40]. By inhibiting glycation, skincare products aim to maintain skin structure and function. The glycation inhibition assay measures the ability of a compound to inhibit glucose‐mediated glycosylation. Naringenin concentrations ranging from 0.02% to 0.13% significantly inhibited glycation compared to solvent controls (Figure 4C). The percent inhibition is concentration‐dependent, indicating that naringenin has anti‐AGE activity overall.

#### 3.2.3 Anti‐Aging Clinical Efficacy of Naringenin Topical Treatment

**FIGURE 5:**
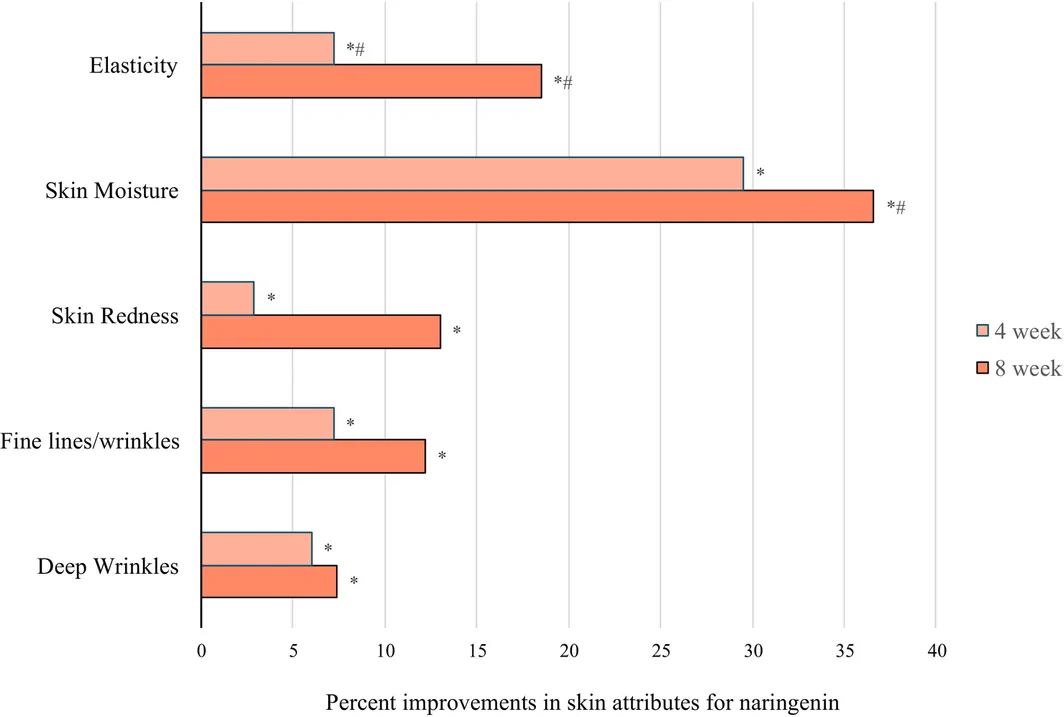
Topical naringenin treatment shows improvement in skin elasticity, moisture, redness, fine lines, and deep wrinkles at four‐ and eight‐week time points. The data shown represent the mean change from baseline for each attribute in the naringenin group of the study (*n* = 34). *p‐value < 0.05, threshold for statistical significance compared to T0. ^#^ *p*‐value < 0.05 threshold for statistical significance vs. placebo.

In addition to skin redness presented above, skin attributes measured by this study included fine lines and wrinkles, deep wrinkles, skin moisture, and skin elasticity. A summary of the outcomes is presented in Figure 5, where naringenin treatment demonstrates improvements in each of the tested skin attributes at the specified time points.

##### 3.2.3.1 Effect of Naringenin on Fine Lines/Wrinkles and Deep Wrinkles

Fine lines and wrinkles were evaluated with the VISIA CR imaging platform. Standardized facial images were captured by a qualified technician, and automated image analysis software was applied to quantify changes in skin surface over time. Reductions in mean area scores indicate improvements in the visual appearance of fine lines and wrinkles. Table 2 presents data on fine lines and wrinkles, including analysis at 4‐week and 8‐week timepoints. For the naringenin subjects, measurements at 4 and 8 weeks compared to baseline showed mean change improvements of 7.2% and 12.2%. These improvements were significant, and 97% of the subjects showed improvement after using naringenin for both 4 and 8 weeks.

**TABLE 2:** Summary of VISIA image evaluation of fine lines/wrinkles.

Article	Time	Mean score ± SD	*p*	Mean change from baseline (%)	Subjects with improvement from baseline (%)
Placebo	Baseline	661 ± 91.2	—	—	—
	4 weeks	647 ± 62.0	0.211	−2.0	55
	8 weeks	644 ± 70.4	0.175	−2.5	52
Naringenin	Baseline	694 ± 139.7	—	—	—
	4 weeks^a^	645 ± 63.6	0.004	−7.2	97
	8 weeks^a^	610 ± 48.5	< 0.001	−12.2	97

^a^ Statistically significant difference from baseline, *p* ≤ 0.05.

**FIGURE 6:**
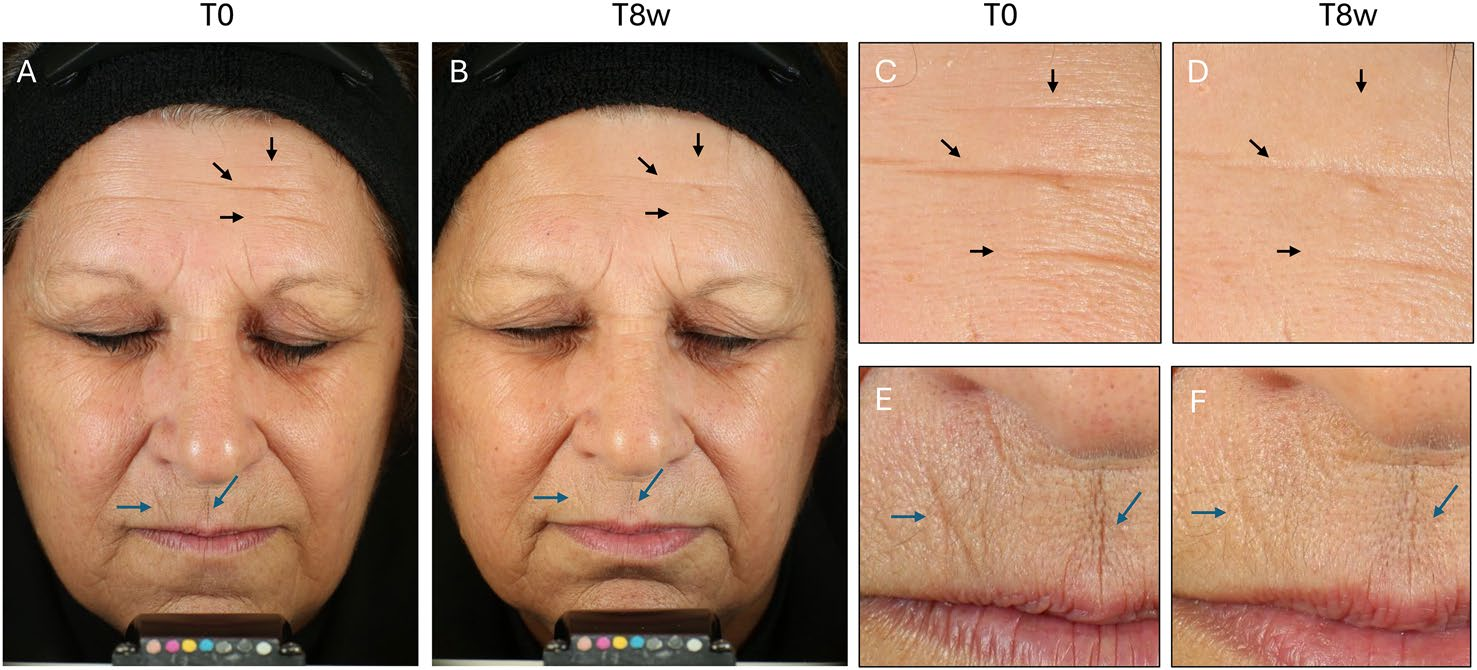
An example clinical study participant demonstrating a reduction in fine lines and wrinkles following naringenin topical treatment. (A) Baseline time zero full face image, and (B) eight‐week timepoint full face image. The images in C and D are a more detailed view of the frontal region with black arrows, and the images in E and F are a more detailed view of the upper lip area with blue arrows, highlighting the differences between T0 and T8 weeks.

**FIGURE 7:**
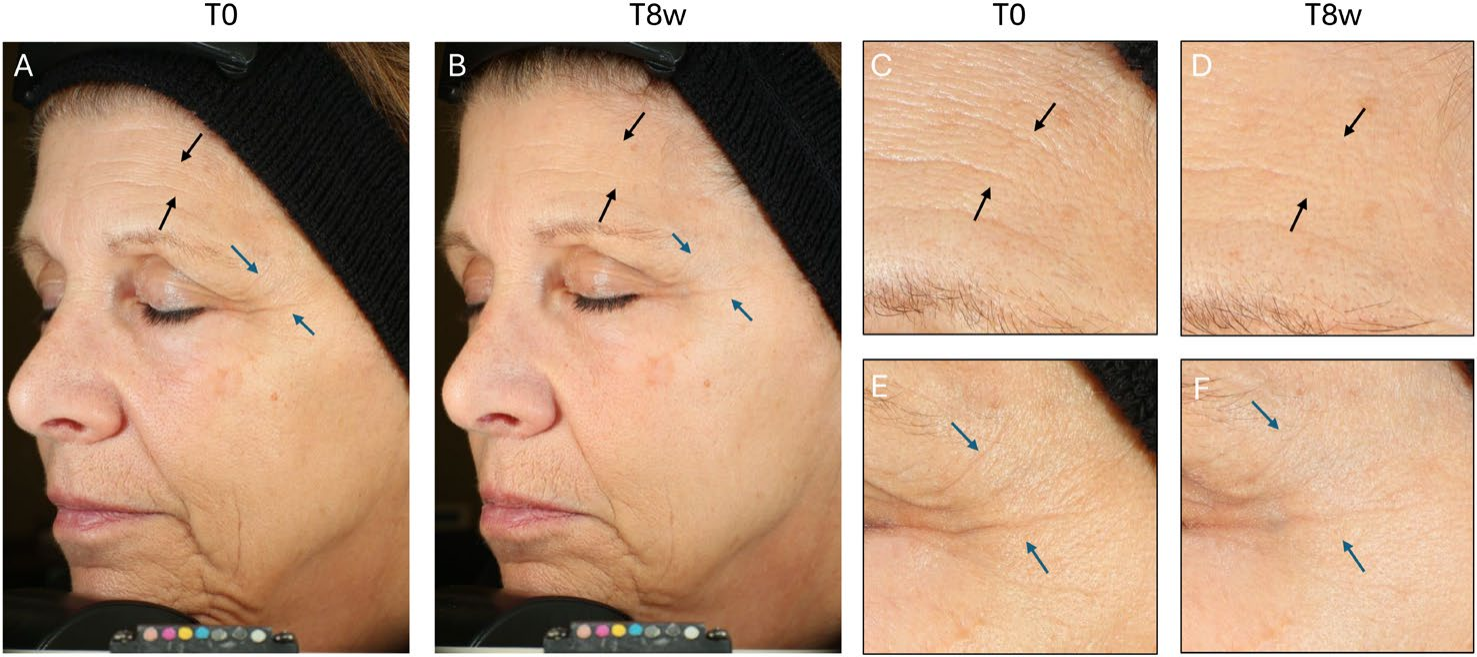
An example clinical study participant demonstrating a reduction in deep wrinkles and crow's feet following naringenin topical treatment. (A) Baseline time zero full face image, and (B) eight‐week timepoint full face image. The images in C and D are a more detailed view of the frontal region, with black arrows, and the images in E and F are a more detailed view of the lateral periorbital region, with blue arrows, highlighting the differences between T0 and T8 weeks.

Deep Wrinkles were scored as an additional skin attribute category using the same VISIA image evaluation as fine lines/wrinkles. Data for deep wrinkles are shown in Table 3. For the naringenin subjects, measurements at 4 and 8 weeks compared to baseline showed mean change improvements of 6.0% and 7.4%. These changes were statistically significant, with 97% of subjects demonstrating improvement at both the 4‐ and 8‐week timepoints following naringenin use. Representative wrinkle reductions are illustrated in Figure 6 and Figure 7. Black and Blue arrows highlight areas of noticeable improvement following naringenin treatment for 8 weeks.

**TABLE 3:** Summary of VISIA image evaluation of deep wrinkles.

Article	Time	Mean score ± SD	*p*	Mean change from baseline (%)	Subjects with improvement from baseline (%)
Placebo	Baseline	405 ± 35.3	—	—	—
	4 weeks	405 ± 39.6	0.942	0	48
	8 weeks	400 ± 37.3	0.117	−1.3	70
Naringenin	Baseline	430 ± 42.0	—	—	—
	4 weeks^a^	404 ± 37.2	< 0.001	−6.0	97
	8 weeks^a^	398 ± 35.8	< 0.001	−7.4	97

^a^ Statistically significant difference from baseline, *p* ≤ 0.05.

##### 3.2.3.2 Effect of Naringenin on Skin Moisture

Skin moisture of the face was measured with a Corneometer, where an increase represents an improvement. Data for both the placebo and naringenin treatments are presented in Table 4, including analysis at 4‐week and 8‐week timepoints. For the naringenin subjects, measurements at 4 and 8 weeks compared to baseline showed mean change improvements of 29.5% and 36.6%. The observed improvements were statistically significant, with 97% of participants showing improvement at 4 weeks and 100% at 8 weeks following naringenin treatment. While both test formulations produced measurable benefits, comparative analysis revealed that the naringenin‐containing product was statistically superior to the placebo at the 8‐week assessment (*p* < 0.001).

**TABLE 4:** Summary of corneometer evaluation.

Article	Time	Mean score ± SD	*p*	Mean change from baseline (%)	Subjects with improvement from baseline (%)
Placebo	Baseline	34.0 ± 4.6	—	—	—
	4 weeks^a^	48.3 ± 9.7	< 0.001	42.1	91
	8 weeks^a^	42.8 ± 12.4	< 0.001	25.9	70
Naringenin	Baseline	38.0 ± 7.3	—	—	—
	4 weeks^a^	49.2 ± 7.3	< 0.001	29.5	97
	8 weeks^a^, ^b^	51.9 ± 6.7	< 0.001	36.6	100

^a^ Statistically significant difference from baseline, *p* ≤ 0.05.

^b^ Statistically significant difference from placebo, *p* ≤ 0.01.

##### 3.2.3.3 Effect of Naringenin on Elasticity

Facial skin elasticity was evaluated using the Cutometer R2 parameter, with higher values indicating greater elasticity. Data for both the placebo and naringenin treatments are presented in Table 5, including analysis at 4‐week and 8‐week timepoints. For the naringenin subjects, measurements at 4 and 8 weeks compared to baseline showed mean change improvements of 7.2% and 18.5%. These improvements were statistically significant, with all participants demonstrating enhanced outcomes at both 4 and 8 weeks of naringenin use. Notably, comparison with the placebo formulation showed that the naringenin‐containing product was statistically superior at both time points (*p* < 0.05).

**TABLE 5:** Summary of cutometer R2 evaluation.

Article	Time	Mean score ± SD	*p*	Mean change from baseline (%)	Subjects with improvement from baseline (%)
Placebo	Baseline	0.498 ± 0.127	—	—	—
	4 weeks	0.461 ± 0.121	0.230	−7.4	31
	8 weeks	0.509 ± 0.122	0.725	2.2	48
Naringenin	Baseline	0.362 ± 0.044	—	—	—
	4 weeks^a^, ^b^	0.388 ± 0.052	< 0.001	7.2	100
	8 weeks^a^, ^b^	0.429 ± 0.060	< 0.001	18.5	100

^a^ Statistically significant difference from baseline, *p* ≤ 0.05.

^b^ Statistically significant difference from placebo, *p* ≤ 0.05.

## 4 Discussion

Skin aging results from the combined effects of extrinsic environmental stressors and the decline of intrinsic biological processes over time, many of which are captured in the established hallmarks of aging [2]. Of particular relevance to the skin is chronic inflammation (inflammaging), which contributes to oxidative stress, promotes cellular senescence, and compromises overall skin function [41].

This study presents evidence that the natural flavonoid naringenin provides multimodal protection against skin inflammaging. Gene expression profiling revealed broad downregulation of transcripts associated with proinflammatory biological processes, notably NF‐κB, a key regulator of cytokine production, including IL‐6 and TNF‐α [42]. These cytokines drive inflammation, oxidative stress, and accelerate skin aging [43]. Our findings align with prior work showing that plant‐derived polyphenols can attenuate inflammation by suppressing multiple signaling pathways [35, 36]. The gene expression changes seen were further validated at the protein level, as our ELISA data confirm significant reductions in proinflammatory cytokines in naringenin‐treated keratinocytes. Our results are in agreement with previous studies on the capacity of naringenin to reduce inflammation in rodents and fibroblast reporter cell lines [25, 26, 44]. Our clinical study demonstrated a visible decrease in skin redness, a hallmark of low‐grade inflammation in aging skin. Together, naringenin modulates gene expression that translates into functional anti‐inflammatory activity in human skin applications.

In addition to its anti‐inflammatory effects, naringenin also modulated several other biological processes that are central to cutaneous aging. In acellular assays, naringenin demonstrated a clear concentration‐dependent radical scavenging activity, consistent with the well‐established antioxidant properties of polyphenolic compounds [45, 46, 47]. Furthermore, inhibition of protein glycation was observed, suggesting that naringenin limits AGE generation. AGE accumulation is known to adversely affect dermal structure by promoting extracellular matrix breakdown, impairing elastin function, and reducing skin hydration, all of which are characteristic of aged skin [48]. Beyond its antiglycation effects, naringenin also suppressed elastase activity in a dose‐responsive fashion. Together, these complementary acellular assay results support the conclusion that naringenin has multiple anti‐aging activities related to skin structure and function.

From a clinical perspective, the biological anti‐aging mechanisms of naringenin result in measurable improvements in skin health. Study participants demonstrated visible softening of wrinkles, improved elasticity, and enhanced skin moisture. These real‐world outcomes align with prior research on plant metabolites, including polyphenols, which have shown an enhancement of skin attributes [49]. Notably, the broad mechanistic profile of naringenin enables simultaneous modulation of inflammatory pathways and multiple contributors to skin aging with a single active compound, in contrast to conventional skincare regimens that rely on combinations of actives such as retinoids, peptides, or vitamins. These data support naringenin as a streamlined and effective strategy for targeting inflammaging in cosmetic dermatology.

## 5 Conclusion

These findings demonstrate that naringenin exerts biological effects relevant to skin inflammaging, supported by complementary mechanistic and clinical evidence. Collectively, the results support the potential of naringenin as a multifunctional cosmetic ingredient that targets several biological processes implicated in skin aging.

## Ethics Statement

The authors confirm that they adhered to the journal's ethical policies, as noted on the journal's author guidelines page. All participants received a thorough explanation of the clinical study and provided written informed consent, including consent to publish their photographs and relevant clinical information. An IRB review was not required.

## Conflicts of Interest

T.O., Z.S., and N.B. are employees of Debut Biotechnology Inc. T.O. is a named inventor on a patent related to the work described in this article, which is owned exclusively by Debut Biotechnology Inc. Debut Biotechnology Inc. provided the test ingredient and participated in the study design, data analysis, data interpretation, and manuscript preparation.

## Supporting information


**Figure S1:** Quantitative ^1^H NMR of naringenin in DMSO‐d6. The sample contains ethylene carbonate as the internal standard (singlet at 4.48 ppm).

## Data Availability

Research data contain proprietary information and cannot be shared due to commercialization restrictions.
